# Optimized Design of EdgeBoard Intelligent Vehicle Based on PP-YOLOE+

**DOI:** 10.3390/s24103180

**Published:** 2024-05-16

**Authors:** Chengzhang Yao, Xiangpeng Liu, Jilin Wang, Yuhua Cheng

**Affiliations:** 1 College of Information, Mechanical and Electrical Engineering, Shanghai Normal University, Shanghai 201418, China; 1000518964@smail.shnu.edu.cn; 2 Shanghai Research Institute of Microelectronics, Peking University, Shanghai 201203, China; wangjl@shrime-pku.org.cn

**Keywords:** PP-YOLOE+, target detection, EdgeBoard intelligent vehicle, Pos-PID, autonomous sensing, optimal route determination

## Abstract

Advances in deep learning and computer vision have overcome many challenges inherent in the field of autonomous intelligent vehicles. To improve the detection accuracy and efficiency of EdgeBoard intelligent vehicles, we proposed an optimized design of EdgeBoard based on our PP-YOLOE+ model. This model innovatively introduces a composite backbone network, incorporating deep residual networks, feature pyramid networks, and RepResBlock structures to enrich environmental perception capabilities through the advanced analysis of sensor data. The incorporation of an efficient task-aligned head (ET-head) in the PP-YOLOE+ framework marks a pivotal innovation for precise interpretation of sensor information, addressing the interplay between classification and localization tasks with high effectiveness. Subsequent refinement of target regions by detection head units significantly sharpens the system’s ability to navigate and adapt to diverse driving scenarios. Our innovative hardware design, featuring a custom-designed mainboard and drive board, is specifically tailored to enhance the computational speed and data processing capabilities of intelligent vehicles. Furthermore, the optimization of our Pos-PID control algorithm allows the system to dynamically adjust to complex driving scenarios, significantly enhancing vehicle safety and reliability. Besides, our methodology leverages the latest technologies in edge computing and dynamic label assignment, enhancing intelligent vehicles’ operations through seamless sensor integration. Our custom dataset, specifically designed for this study, includes 4777 images captured by intelligent vehicles under a variety of environmental and lighting conditions. The dataset features diverse scenarios and objects pertinent to autonomous driving, such as pedestrian crossings and traffic signs, ensuring a comprehensive evaluation of the model’s performance. We conducted extensive testing of our model on this dataset to thoroughly assess sensor performance. Evaluated against metrics including accuracy, error rate, precision, recall, mean average precision (mAP), and F1-score, our findings reveal that the model achieves a remarkable accuracy rate of 99.113%, an mAP of 54.9%, and a real-time detection frame rate of 192 FPS, all within a compact parameter footprint of just 81 MB. These results demonstrate the superior capability of our PP-YOLOE+ model to integrate sensor data, achieving an optimal balance between detection accuracy and computational speed compared with existing algorithms.

## 1. Introduction

Designed to elevate the efficiency and safety of urban transport, EdgeBoard intelligent vehicles increasingly underpin the development of smart cities amid the swift advance of intelligent transportation and autonomous driving technologies [1]. Ensuring efficient and safe operation necessitates that these vehicles are equipped with precise and rapid environmental perception. This requires onboard systems capable of not only real-time, accurate object identification and localization but also dependable performance in dynamic environment.

This work conducts a comprehensive analysis of the PP-YOLOE [2] architecture and introduces enhancements to meet the distinct needs of traffic environments. The PP-YOLOE+ model, an advancement of the original framework, adopts sophisticated feature extraction and optimization algorithms. It first enhances small and blurred object recognition in complex scenes through an improved feature extraction network. It then employs a refined multiscale detection approach to heighten detection precision for objects of diverse sizes. Additionally, with the real-time demands of intelligent vehicles in mind, the model’s computational efficiency has been optimized to deliver rapid processing while preserving recognition accuracy.

Modern deep-learning-based detection algorithms directly extract features from raw data, markedly enhancing the efficiency and accuracy of detection tasks. These algorithms fall into two main categories. The first includes two-stage methods, noted for their precision but associated with higher computational demands. The second category consists of one-stage methods, which are preferred for their rapid processing speeds and straightforward implementation. Despite their slightly lower accuracy, the simplicity and wide applicability of one-stage methods have made them extremely popular in practical settings. Furthermore, researchers are actively refining existing deep learning frameworks to meet the critical demand for efficient and accurate object detection algorithms in the intelligent vehicle sector.

By integrating advanced technologies, our work has not only improved the model but also optimized the design for the EdgeBoard intelligent vehicle system. We have integrated the sophisticated PP-YOLOE+ model with the vehicles’ perception and decision-making algorithms to enhance the detection accuracy of small and dynamic targets in traffic environments. Notably, the integration of EdgeBoard’s efficient edge computing devices facilitates the real-time execution of complex algorithms, significantly reducing energy consumption and latency. Experiments conducted across various traffic scenarios thoroughly assess the reliability and performance of the enhanced PP-YOLOE+ model and EdgeBoard intelligent vehicles under real-world conditions. These improvements enable EdgeBoard intelligent vehicles to achieve greater accuracy and real-time performance in complex urban traffic settings, markedly boosting urban transportation safety and efficiency.

## 2. Related Work

Recent advancements in deep learning have significantly propelled the domain of traffic object detection forward. By leveraging the power of neural networks to extract features directly from raw data, deep learning-based detection algorithms have markedly enhanced both efficiency and accuracy. These algorithms are broadly classified into two types: two-stage methods, which prioritize candidate regions and are notable for their precision, including mask region convolutional neural networks (Mask R-CNNs) [3], spatial pyramid pooling networks (SPPNets) [4], Fast R-CNNs [5], Faster R-CNNs [6], and region-based fully convolutional networks (R-FCNs) [7], though with considerable computational complexity; and one-stage methods, characterized by their simplicity, speed, and broader applicability but with somewhat lower accuracy, encompassing approaches such as single-shot detectors (SSDs) [8], YOLO [9], YOLOv2 [10], YOLOv3 [11], and YOLOv4 [12].

### 2.1. Two-Stage Detection Methods

With the evolution of intelligent transportation systems and the advent of deep learning, particularly in the intelligent vehicle domain, there has been a significant surge in demand for efficient and accurate object detection algorithms. While models grounded in conventional machine vision offer simpler implementation, researchers are now advancing these through the optimization of existing deep learning frameworks. Currently, the use of convolutional neural networks (CNNs) in traffic-related applications is becoming more prevalent. By exploiting features such as appearance, motion patterns, and spatial layout within images, CNNs are capable of efficiently detecting and recognizing complex traffic scenarios. These methods excel in enhancing the accuracy and real-time capabilities of vehicle detection, offering a competitive edge in intelligent transportation systems. Sanjay et al. [13] investigated a CNN-based approach for training classifiers for both multiclass and single-class object detection, including their application on Android devices. By merging SSD architecture with MobileNets, this method achieved a balanced image processing outcome, enhancing processing speed and detection rate, though it increased computational complexity. Mikic et al. [14] proposed a segmentation algorithm for traffic scenes capable of distinguishing moving objects from shadows with greater accuracy, employing color, neighborhood, and temporal data, though it also used grayscale and depth images for detecting various targets. These innovations harness advanced deep learning and image processing algorithms to elevate the performance of traffic monitoring systems, incorporating techniques like attention mechanisms, LSTM, dynamic region amplification, and sophisticated feature extraction to enhance detection in complex environments, particularly under low-light and nighttime conditions. The accuracy of the target location is significantly enhanced in scenes with long shadows when integrated with smart cars. However, the practical application of these methods faces challenges due to the demands for high-performance computing resources, especially for complex algorithms, and their performance can be impacted by extreme weather conditions, peculiar lighting, and noise. Despite improvements, the detection accuracy for very small or fast-moving objects may remain constrained. Moreover, the extensive data required for training and the subjective nature of feature extraction to achieve optimal performance add to the complexity of implementation, affecting the robustness of the models.

The development of CNN-based [15] technologies has rapidly advanced in recent years. Compared with traditional approaches, CNNs extract features at various levels from input images, facilitating precise object detection through information classification and positional regression. Parmar and his team [16] have refined convolutional neural networks (CNNs) by incorporating a range estimation layer, enabling the simultaneous detection, classification, and ranging of objects. After combining with intelligent vehicles, in the highway scene, the ranging error of automatic driving is greatly reduced in the highway scene, and the distance of objects can be distinguished in time. Despite its innovative potential, creating a robust detection and ranging system for real-world deployment presents challenges, particularly due to safety concerns across varied lighting and weather conditions. Oh [17] and colleagues unveiled an innovative approach for object detection and classification within driving contexts by employing a decision-level fusion of CNN-based classifiers on 3D point clouds and image data. This method notably surpassed previous strategies in precision, according to the KITTI benchmark dataset, for identifying cars, pedestrians, and cyclists and it significantly enhanced the performance and reliability of intelligent vehicle systems. Meyer [18] demonstrates the effectiveness of using deep convolutional neural networks for 3D object detection by comparing deep learning methods on radar point clouds with camera images, where radar data outperforms lidar, although currently the main limiting factor in performance is the size of the dataset. This improvement is particularly critical in complex driving environments where the integration of radar and camera data helps to overcome the limitations of each sensor alone, thereby increasing the overall safety and operational effectiveness of autonomous vehicle systems. Aradhya [19] crafted CNN models for the detection of single and multiple objects in urban vehicle datasets, gauging their performance via metrics like TP, TN, FP, FN, accuracy, confusion matrix, and *mAP*. Integrating YOLOv3 with SORT for efficient cross-frame object tracking in traffic surveillance, this method utilizes the powerful features of networks like DarkNet. This combination ensures real-time, accurate, and precise vehicle identification, which is critical for effective traffic management applications. However, the robustness of these models requires enhancement due to the scarcity of images. In addition, Fang [20] improves the Mask R-CNN framework for the perception of autonomous driving environments by integrating the ResNeXt network and group convolution to enhance feature extraction. This enhancement includes adding a bottom-up path enhancement and an effective channel attention module to the framework, as well as substituting the smooth L1 loss with CIoU loss for enhanced model convergence and precision. The refined algorithm exhibited considerable improvements in detection and segmentation precision across the CityScapes and BDD datasets, proving its efficiency in intricate traffic situations and it ensures better environmental awareness and safety in dynamic driving conditions. Another study introduces an innovative integrated multimodal fusion deep neural network (IMF-DNN) framework, aimed at object detection and comprehensive driving strategies, as proposed by Nie et al. [21]. They also devised a DNN safety testing strategy, focusing on systematically analyzing the robustness and generalization of DNNs in various driving conditions, thereby enhancing the performance of deep learning models for autonomous driving. While small networks show potential in embedded systems, their robustness and accuracy require further improvement. Mahmood and his team [22] introduced an improved automatic license plate detection (ALPD) method for intelligent transport systems, combining Faster R-CNN with digital image processing techniques for precise detection of license plates. Utilizing color segmentation, morphological filtering, and size analysis in a license plate localization module (LPLM), this approach attains notable accuracy and efficiency on the PKU datasets, demonstrating its potential for security and target identification purposes. When combined with intelligent vehicles, the proposed model yields higher detection accuracy in a shorter execution time.

### 2.2. Single-Stage Detection Methods

By merging SSD architecture with MobileNets, this method achieved a balanced image processing outcome, enhancing processing speed and detection rate, though it increased computational complexity. Li and associates [23] introduced YOLOv4_Drone, an augmented detection model incorporating hollow convolutions, an ultralightweight subspace attention mechanism (ULSAM), and soft nonmaximum suppression (Soft-NMS), improving detection in cluttered backgrounds and occlusion scenarios, limited by computational resources. Tao [24] proposed OYOLO, an optimized YOLO variant that integrates R-FCN and employs histogram equalization for preprocessing nighttime images, achieving superior speed and accuracy, notably in demanding nighttime traffic scenes. Significant improvements in the accuracy and speed of real-time object recognition at night are achieved when this technology is integrated with intelligent vehicles, enhancing both safety and navigation efficiency in autonomous driving systems. Wang et al. [25] enhanced the SSD model to create AP-SSD, utilizing multishape Gabor feature extraction, Bottle Neck-LSTM for interframe information correlation, and dynamic region magnification, significantly boosting detection precision and efficiency in complex traffic scenarios. Integration with intelligent vehicles significantly enhances the recognition accuracy of autonomous driving systems, especially when encountering small objects, multiple objects, cluttered backgrounds, or large-area occlusions. Addressing traffic congestion in Macau, Lam et al. [26] developed a low-cost, real-time traffic monitoring system using free online images, YOLOv3, and the mIOU algorithm, which showed high accuracy and adaptability in diverse conditions. Ye [27] presents the vehicle-based efficient low-light image enhancement (VELIE) network, utilizing the Swin Vision Transformer combined with a gamma transformation enhanced U-Net. This approach aims to improve low-light images, overcoming the challenges faced by RGB cameras in advanced driving assistance systems (ADAS). It offers an economical and high-performance option, achieving rapid processing in just 0.19 s for enhanced nighttime environmental awareness. Qiu and colleagues [28] have introduced IDOD-YOLOv7, a framework that combines image dehazing (AOD) and image enhancement (SAIP) to improve target detection performance under low-light and foggy conditions for autonomous driving. This approach, by creating a specialized dataset for low-light and foggy traffic images (FTOD) and conducting end-to-end joint learning, effectively increases the model’s detection accuracy and robustness under complex weather conditions. However, achieving ideal results in practice can be challenging due to the limited precision of parameters. Additionally, Sudha and colleagues [29] have developed “enhanced you only look once v3”, an innovative deep learning approach that integrates an improved visual background extractor for the precise detection of various vehicle types and numbers in videos. Leveraging the Kalman filter and particle filtering techniques for tracking, their methodology was tested under a range of weather conditions, showing high accuracy and tracking effectiveness of up to 96.6% in sunny, rainy, nighttime, and foggy scenarios. After integrating with intelligent vehicles, the system effectively estimates the target area’s information through various vehicle detection and tracking methods and gathers data for the vehicle detection model. Concurrently, it collects diverse datasets from multivehicle detection and tracking. This method enhances the robustness and efficiency of monitoring systems in autonomous vehicles by accurately identifying and continuously tracking multiple vehicles under diverse traffic conditions. Consequently, it equips autonomous driving systems with improved situational awareness and decision-making capabilities, ensuring safer and more reliable navigation through complex environments. However, the substantial computational demand for processing extensive data highlights the significant need for computing resources. Additionally, Cao et al. [30] introduced an advanced vehicle detection method for intelligent vehicles, enhancing the SSD model through optimization. This improvement encompassed the model’s architecture, training technique, and loss function, resulting in a notable mean average precision (*mAP*) of 92.18% and an average processing time of 15 milliseconds on the KITTI dataset. This achievement denotes significant advancements in precision, real-time capabilities, and adaptability to challenging conditions and harsh weather, offering essential assistance for the effective functioning of intelligent vehicles in actual traffic situations. The YOLO algorithms have consistently faced challenges in detecting small objects. To address this issue, Qu [31] developed the inaugural PP-YOLOE algorithm specifically designed for small targets. By integrating a coordinate attention mechanism and an optimized feature pyramid structure into the algorithm’s backbone, they significantly enhanced the accuracy and speed of small object detection, demonstrating substantial practical value in industrial applications. Zhang [32] introduced an enhanced PP-YOLOE-m network that markedly improved the accuracy and speed of surface defect detection in strip steel. By incorporating data augmentation, coordinate attention technology, and advanced spatial pyramid pooling, the model achieved significant performance gains on the NEU-DET dataset. In summary, the methods used for object detection within intelligent transportation systems must achieve an optimal balance among complex environmental factors, accuracy, and speed of detection. See Table 1.

## 3. The PP-YOLOE+ Model

### 3.1. Backbone Network

This paper proposes the PP-YOLOE+ model for object detection and recognition, which, due to its reduced computational requirements and low latency, is well suited for integration into intelligent vehicle systems.

**Figure 1:**
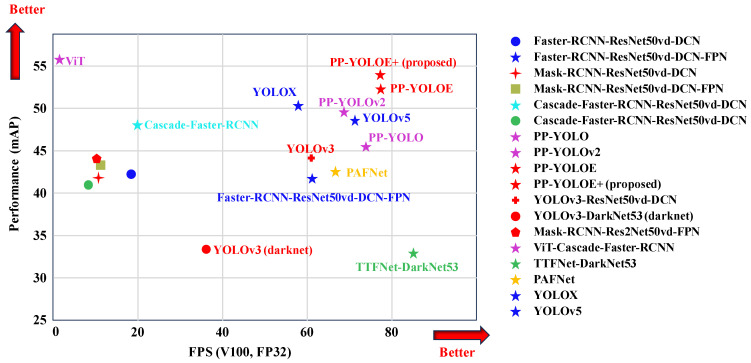
Illustration of the relationship between the accuracy metrics of the mobile model and its variation in prediction time.

The PP-YOLOE network was proposed by Xu et al. [2], and PP-YOLOE is an enhanced YOLO object detection model featuring a powerful CSPRepResStage backbone and an efficient task-aligned head (ET-head) with optimizations like dynamic label assignment and improved inference speeds, making it highly effective for real-time applications, so our PP-YOLOE+ is characterized by a low-latency structure, while offering high speed and accuracy. Table 2 provides a detailed comparison of their structures. It outperforms similar parameter models such as YOLOv3 [33] and YOLOv5 [34], as shown in Figure 1. This network enhances standard convolution and incorporates hyperparameters that simplify the network architecture, thereby reducing parameters and computational requirements, as well as accelerating network training speed.

*AP*, or average precision, measures the precision of a model as a function of recall in computer vision, evaluating how accurately the model identifies objects. It is calculated for each class and at different recall levels. Following this, *mAP*, or mean average precision, aggregates these *AP* scores to provide a comprehensive performance metric. *mAP* represents the mean of the *AP* values calculated across all classes or various recall thresholds. This metric offers a single figure summarizing the precision–recall performance of a model across different categories or thresholds, establishing it as a standard benchmark in tasks like object detection and segmentation. Higher *mAP* values indicate superior model performance, especially in terms of accurately identifying objects across different classes with high confidence. The definitions of *AP* and *mAP* are as follows:

(1) $$AP=\sum_{n}\left(Rec_{n}-Rec_{n-1}\right)\times Pre_{n}.$$

(2) $$mAP=\frac{1}{N}\sum_{i=1}^{N}AP_{i}.$$

**Figure 2:**
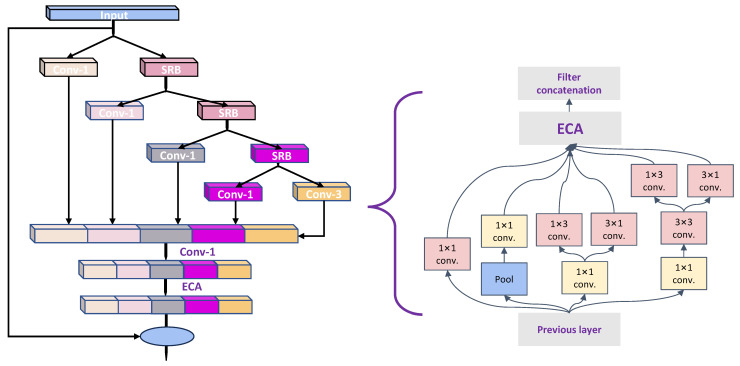
The structure of RepResBlock.

**Figure 3:**
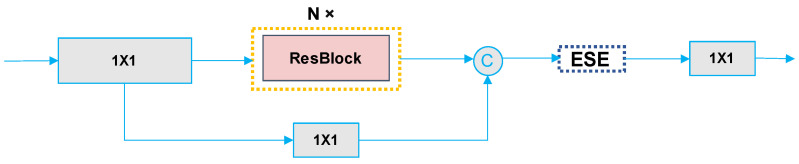
The structure of the model following the integration of RepResBlock into the stage.

Taking inspiration from TreeBlock [35], PP-YOLOE+ implements the innovative RepResBlock, which combines residual and dense connections within its backbone and neck, leading to a significant accuracy enhancement of 0.7 *mAP*, as illustrated in Figure 2 and Figure 3.

In our backbone design, diverging from ResNet, we initially adopted a series of three consecutive convolutional layers as the stem. Subsequently, we introduced the RepResBlock structure, detailed in Figure 3 above. Furthermore, each stage was augmented with an ESE module and the integration of residual connections.

The strategy for assigning labels is crucial in object detection, with PP-YOLOE+ utilizing the SimOTA assignment strategy. Task alignment learning (TAL) is built upon dynamic label assignment and task alignment loss. This dynamic assignment is predicated on prediction sensitivity, dynamically allocating positive samples to each ground truth target based on their predictions. TAL, by deliberately synchronizing these two tasks, is capable of simultaneously attaining optimal classification accuracy and the utmost precision in bounding box determination. TAL has been shown to enhance accuracy by 0.9 *mAP*.

**Figure 4:**
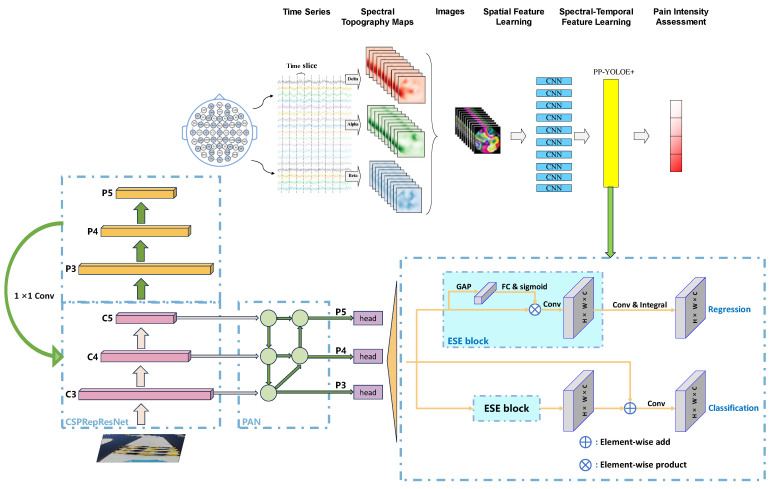
Structure of PP-YOLOE+.

To mitigate the conflict between classification and localization tasks, PP-YOLOE+ deploys the efficient task-aligned head (ET-head) structure, achieving an accuracy enhancement of 0.5 *mAP*. The comprehensive network architecture is illustrated in Figure 4.

### 3.2. Parameter Optimization for the PP-YOLOE+ Model

(1) Batch size: Batch size is notably the simplest hyperparameter to adjust, often being the initial choice made. Thus, determining a suitable batch size is a prerequisite to adjusting other hyperparameters. The optimal size hinges on maximizing GPU memory utilization (utilization can be monitored using the terminal command nvidia-smi), which implies selecting the largest viable batch size. Enlarging the batch size refines the descent trajectory, reducing training fluctuations. While fully leveraging the GPU accelerates training, it demands more iterations to achieve comparable accuracy, given the reduced frequency of parameter updates per epoch. In the context of fine-tuning, a larger batch size can foster improved network convergence and diminish the need for alternative regularization methods.

(2) Learning rate: The preset learning rate within the paddle detection configuration is designed for multi-GPU setups (commonly 8 GPUs, with PicoDet utilizing 4). When shifting to single-GPU training, an adjustment of the learning rate is imperative, necessitating division by 8. Correspondingly, batch size adjustments should be made in a proportional manner. From this, the subsequent formula is deduced:

(3) $$\frac{Lr_{0}}{bs_{0}\times n_{0}}=\frac{Lr}{bs\times n}.$$

In the original configuration, $Lr_{0}$ signifies the learning rate, $bs_{0}$ indicates the batch size, and $n_{0}$ quantifies the GPUs utilized. This research accelerates training convergence and enhances model efficacy by applying a stochastic gradient descent strategy for alternating model training. An initial learning rate is established at 0.001, with a momentum parameter set at 0.9 to guide the learning process. The network undergoes 80 training iterations at this starting rate.

(3) Multiscale training [36]: Input image dimensions crucially affect detection model performance. Multiscale strategies are notably effective in improving accuracy. The feature maps produced during processing by the base network are substantially smaller than the original image, diminishing the network’s ability to capture features of smaller objects. Incorporating training with images of larger and varied sizes can enhance the model’s robustness in detecting objects across different scales. Despite previous advice against being overly cautious with scale jitter, memory usage constraints (to maintain a reasonable batch size) meant that the increase in size scales for multiscale training was moderate. Consequently, the batch size for training the L model remains limited to approximately 8.

Configuring the network to deliver robust predictive performance for diverse input sizes enables detection at varying resolutions within the same architecture. Processing speeds increase with smaller input image sizes, whereas larger dimensions yield improved accuracy.

(4) DlouLoss: Due to the inability of IOU and GIOU loss functions to precisely determine the relative positions between the actual and predicted bounding boxes (with IOU and GIOU values being identical when the target box entirely encompasses the predicted box, thus failing to differentiate their relative locations), DIoU loss was introduced. The formula for its calculation is presented as follows:

(4) $$DIoU=IoU-\frac{\rho^{2}\left(b,b^{gt}\right)}{c^{2}}.$$

where *b* and $b^{gt}$ correspond to the centroids of the predicted and ground truth boxes, respectively. The symbol $\rho^{2}$ is used to express the Euclidean distance between these centroids. Meanwhile, $c^{2}$ quantifies the diagonal distance across the minimal enclosing rectangle that covers both the predicted and actual boxes. By directly minimizing the distance between the bounding boxes, DIoU loss achieves convergence significantly faster than GIoU loss.

(5) Large-scale testing: Employing bigger scales for testing is intended to enhance the identification of smaller items. Incorporating larger scales in the testing stage accentuates the features of small objects, thus facilitating their detection. It is critical to recognize, however, that a higher image resolution is not invariably advantageous; an escalation in resolution beyond a specific threshold may diminish the accuracy in identifying larger and medium-sized entities.

## 4. Design and Implementation of Intelligent Vehicle Model Based on EdgeBoard

### 4.1. Overview of System Structure

**Figure 5:**
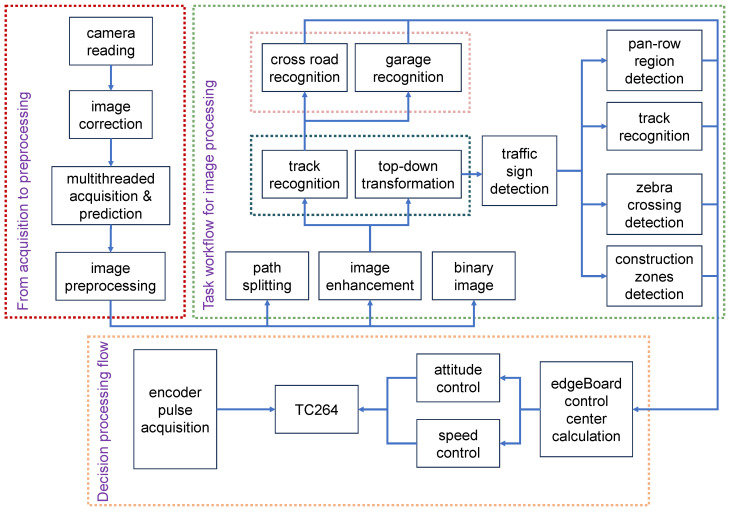
Flowchart of model deployment and inference process.

The system integrates Baidu EdgeBoard for tracking information and element detection and leverages an Infineon TriCore architecture-based AURIX series TC264 microcontroller for motion control. It captures road and signage data through a camera, processes the data using EdgeBoard, and forwards them to the Infineon TC264 via a serial port to trigger appropriate responses. The intelligent cars’ movement is controlled by a Pos-PID closed-loop control algorithm. For further adjustments, auxiliary debugging is facilitated through buttons, display screens, and additional instruments. The workflow for model deployment and inference is depicted in Figure 5.

### 4.2. Comprehensive Hardware Design of the System

**Figure 6:**
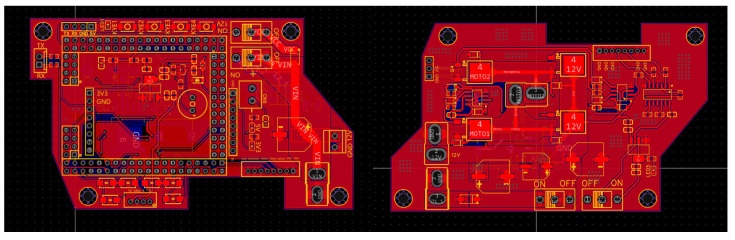
Diagrams of the mainboard (**left**) and drive board (**right**).

Upon program execution, the system initially omits frames with unstable images before the vehicle departs the garage. Subsequently, a grayscale camera deploys a line-tracking algorithm. Following a second passage over the zebra crossing, the vehicle re-enters the garage, and the hardware configuration is detailed in Figure 6.

### 4.3. Designing Algorithmic Control for EdgeBoard-Integrated Intelligent Vehicle Systems

We optimize the Pos-PID [37] algorithm for servo steering control and the ADRC algorithm to regulate the motor speed at a designated target. In the realm of practical engineering, proportional, integral, and differential (Pos-PID) control, also known as Pos-PID tuning, predominates. Pos-PID controllers have emerged as a principal technology in industrial control, acclaimed for their stability, simplicity, reliability, and ease of tuning. These controllers prove indispensable when the controlled object’s structure and parameters are ambiguous, or when a precise mathematical model is unattainable, challenging the application of other control theories. Under such circumstances, the design and parameters of the system’s controller rely on empirical expertise and field tuning, highlighting the utility of Pos-PID technology. This makes Pos-PID control the preferred approach when dealing with incomplete system understanding or when system parameters are not readily measurable. Although PI and PD controls are utilized as well, the Pos-PID controller, a linear controller, produces its control action by linearly blending the errors in proportion (P), integration (I), and differentiation (D), according to the difference between desired and actual output values.

Within digital control systems, a digital PID controller is employed, functioning based on the following control concept:

(5) e(k)=r(k)−c(k).

(6) $$u\left(k\right)=K_{P}\left\{e\left(k\right)+\frac{T}{T_{I}}\sum_{j=0}^{k}e\left(j\right)+\frac{T_{D}}{T}\left[e\left(k\right)-e\left(k-1\right)\right]\right\}.$$

where the index *k* represents sequential sampling instances, incrementing as 0, 1, 2, etc., to mark discrete evaluation or adjustment moments. *r*(*k*) signifies the target value at each *k* instance, serving as the system’s output goal. *c*(*k*) captures the actual input value at the same instance, reflecting the real-world inputs received. The control output *u*(*k*) is the system’s response aimed at addressing the discrepancy from the target value. The deviation *e*(*k*) quantifies the gap between the target *r*(*k*) and actual input *c*(*k*) at each instance, indicating the error that the system aims to minimize. The previous deviation *e*(*k −* 1) provides a basis for comparing error changes over time. The proportional coefficient $K_{P}$ influences the system’s immediate reaction to the current error; $T_{I}$, the integral time constant, impacts the cumulative error correction over time; and $T_{D}$, the differential time constant, affects the system’s predictive response to error changes. Lastly, *T* denotes the sampling period, establishing the interval between consecutive observations and adjustments, thus dictating the system’s response cadence.

To distill, the functionalities of a PID controller [38] components are delineated as follows:

The proportional element mirrors the control system’s deviation signal both promptly and in proportion. Upon the emergence of deviation, it immediately enacts a control measure aimed at diminishing the deviation. The integral aspect is primarily used to eliminate steady-state error, thus bolstering the system’s precision. The effectiveness of the integral action varies with the integral time constant, which means a higher constant weakens the integral effect, while a lower one strengthens it. The differential portion gauges the deviation signal’s rate of alteration, preemptively introducing a corrective signal to the system before the deviation escalates, thus speeding up the system’s responsiveness and reducing the adjustment time. Consequently, digital PID control methods are divided into Pos-PID control algorithms and Roc-PID control algorithms [39].

#### 4.3.1. Pos-PID Controller

In the Pos-PID controller framework, the output *u*(*k*) serves to directly manipulate the actuator, with *u*(*k*)s’ value accurately mirroring the actuator’s position, thereby defining the Pos-PID control algorithm. A notable limitation of this algorithm is its dependence on complete output quantities, tying each output to previous states and necessitating the summation of past errors *e*(*k*), which amplifies the computational burden. Furthermore, since the output *u*(*k*) precisely maps to the actuator’s position, any malfunction within the computing system that significantly shifts *u*(*k*) can cause marked changes in the actuator’s location. Such pronounced shifts are generally untenable in industrial operations, potentially causing serious accidents. This situation has catalyzed the evolution of the Roc-PID control algorithm, distinguished by the digital controller’s output being simply the incremental change, ∆*u*(*k*), in the control variable, to address these concerns.

#### 4.3.2. Roc-PID Controller

When the actuator necessitates the increment of the control quantity, this can be derived from Formula (4), leading to an incremental PID control equation. By deducing Formula (3) from Formula (5), and then subtracting Formula (5) from Formula (4), we obtain Formulas (6) and (7):

(7) $$u\left(k-1\right)=k_{P}\left\{e\left(k-1\right)+\frac{T}{T_{I}}\sum_{j=0}^{k-1}e\left(j\right)+\frac{T_{D}}{T}\left[e\left(k-1\right)-e\left(k-2\right)\right]\right\}.$$

(8) $$\Delta u\left(k\right)=k_{P}\left\{\left[e\left(k\right)-e\left(k-1\right)\right]+\frac{T}{T_{I}}e\left(k\right)+\frac{T_{D}}{T}\left[e\left(k\right)-2e\left(k-1\right)+e\left(k-2\right)\right]\right\}.$$

(9) $$\Delta u\left(k\right)=K_{P}\Delta e\left(k\right)+k_{I}e\left(k\right)+K_{D}\left[\Delta e\left(k\right)-\Delta e\left(k-1\right)\right].$$

where Δe(k) is e(k)−e(k−1), $k_{I}$ representing $k_{P}$$\frac{T}{T_{I}}$ and $K_{D}$ representing $k_{P}$$\frac{T_{D}}{T}$.

Formula 6 presents the Roc-PID control algorithm. It calculates the control increment using deviations from the last three measurements, based on a fixed sampling period *T* typical in control systems, with *KP*, *TI*, and *TD* constants. This algorithm has multiple advantages:

(1) It produces incremental outputs, thus minimizing the impact of incorrect operations, which can be deactivated through logical decisions if needed.
(2) The transition between manual and automatic modes is smooth, promoting seamless switches. Moreover, in case of a computer failure, the output channel or the actuator’s capacity to latch signals preserves the initial value.
(3) The algorithm does not necessitate cumulative calculations. The control increment Δ*u*(*k*) is determined solely by the latest *k* sample values, facilitating improved control quality via weighted methods.

Yet, the Roc-PID controller has shortcomings: it is prone to significant integral wind-up, leading to persistent errors, and is greatly affected by overflow. Consequently, refined PID control strategies that include dead zones and integral separation are often employed to mitigate these drawbacks.

## 5. Experimental Results and Analysis

### 5.1. Datasets

**Figure 7:**
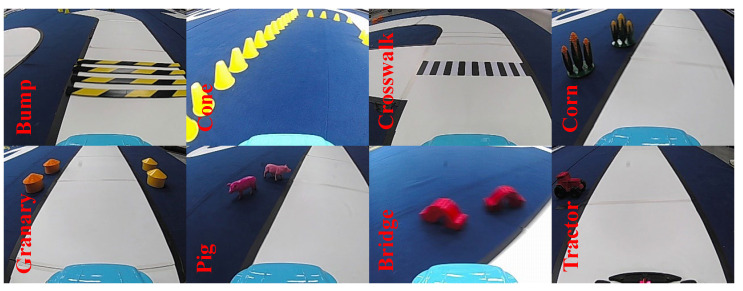
Different kinds of targets in the dataset.

In this work, objects identified and detected are divided into eight categories, as illustrated in Figure 7. Conventional object detection approaches are plagued by inadequate accuracy and slow detection rates. To address these issues, the authors adopted a CNN-based detection algorithm, specifically employing the PP-YOLOE+ from the paddle detection [40] suite. The PP-YOLOE+ framework makes use of the CSPNET [41] convolutional neural network and CSPPAN for feature fusion. Enhancements to the PP-YOLOE+ architecture were made by substituting CSPNET with CSPRepResNET, thereby improving the effectiveness of object detection and recognition.

Datasets play a critical role in deep learning, effectively determining the upper limits of model performance. Thus, initial efforts should concentrate on data analysis to inform precise preprocessing and adjustments to model parameters.

**Figure 8:**
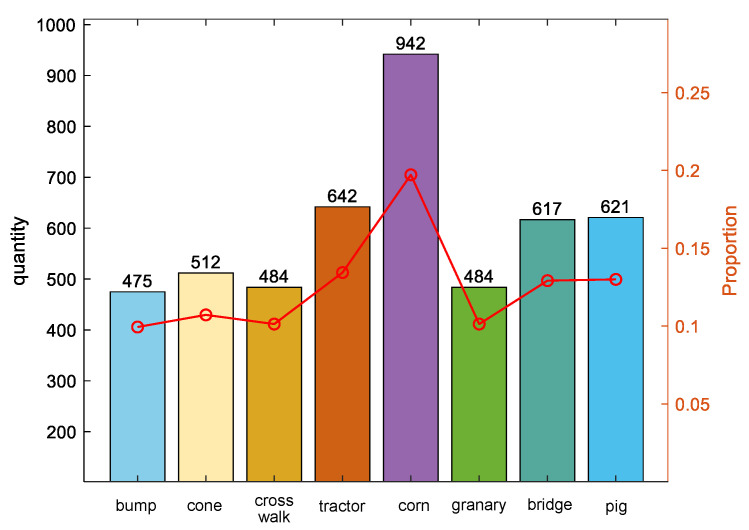
Partition of the dataset.

A dataset comprising 4777 photographs from the perspective of intelligent vehicles was curated and segmented into training and testing sets. Notably, our analysis has identified significant imbalances in the occurrence of different categories, with objects like pedestrians and traffic signs appearing more frequently than rarer items such as animals or construction equipment. Furthermore, there are substantial variations in the sizes of objects within the images, which reflect the real-world situation where objects of interest vary in dimension depending on their distance from the camera. Addressing these issues of category imbalance and size variation will be the primary focus of our ongoing efforts to refine the model, aiming to enhance its accuracy and robustness across different driving conditions. See Figure 8.

### 5.2. Model Evaluation Metrics

Evaluation metrics for image recognition prominently feature accuracy (*A_cc_*) and error rate ($E_{RR}$) as benchmarks for classifying the accuracy of target identification. The error rate reflects the proportion of incorrectly identified samples out of the total, whereas accuracy indicates the fraction of samples correctly identified from all samples. Collectively, the error rate and accuracy sum to one. The definitions of *TP* (true positives), *TN* (true negatives), *FP* (false positives), and *FN* (false negatives) are utilized to differentiate between the various outcomes of recognition precision:

- *TP* (true positives) represents the number of positive samples accurately identified as positive.
- *TN* (true negatives) describes the number of negative samples accurately identified as negative.
- *FP* (false positives) marks the number of negative samples incorrectly identified as positive.
- *FN* (false negatives) signifies the number of positive samples incorrectly identified as negative.

The calculations for accuracy ($A_{cc}$), error rate ($E_{RR}$), precision, recall, and *F*1-*score* are presented through specific formulas:

(10) $$A_{cc}=\frac{TP+TN}{TP+TN+FP+FN}.$$

(11) $$E_{RR}=\frac{FN+FP}{TP+TN+FP+FN}.$$

(12) $$Pre=\frac{TP}{TP+FP}.$$

(13) $$Rec=\frac{TP}{TP+FN}.$$

(14) $$F1-score=\frac{2Pre\times Rec}{Pre+Rec}.$$

In tasks of object detection, intersection over union (*IoU*) stands out as a crucial metric, capturing the ratio of overlap between the model’s suggested bounding box and the actual annotation. Mathematically, it is defined as the ratio between the intersection of the *detected result* and the *ground truth* to their union, as follows:

(15) $$IoU=\frac{Detection\;Result\cap Ground\;Truth}{Detection\;Result\cup Ground\;Truth}$$

### 5.3. Tests for Detecting Targets on the Road

**Figure 9:**
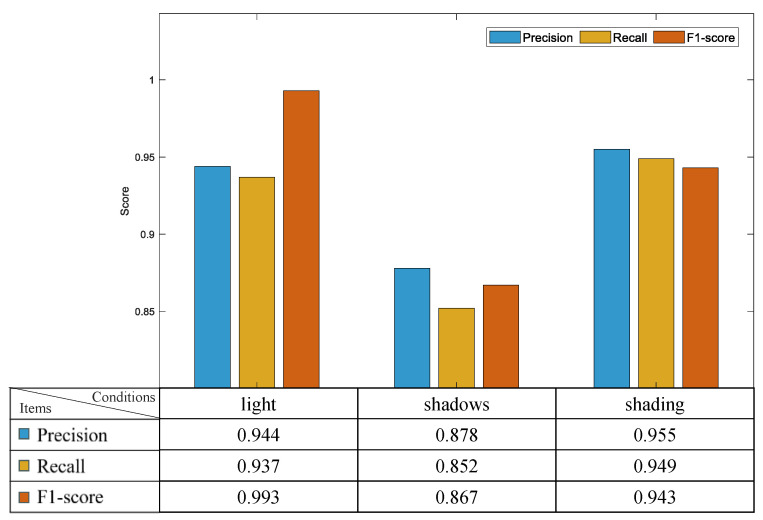
Performance of the PP-YOLOE+ model under different conditions.

Figure 9 outlines the efficacy of the model presented across different lighting conditions, including light, clear with shadows, and shade. Optimal conditions are presented during daylight, where the combined influence of substantial natural and artificial light sources provides consistent illumination. In these conditions, the model attains a precision, recall, and *F*1-*score* of 0.944, 0.937, and 0.993, respectively. However, in shadowy settings, recall and *F*1-*score* are observed at 0.952 and 0.867, respectively. A notable precision surplus of 0.026 over recall is observed, attributable to the color resemblance between cones and their backdrop under shadows, leading to an elevated count of false negatives. The fluctuation in lighting and shadows could result in feature deterioration, with accuracy affected by an increment in false negatives due to diminishing light quality, primarily contributing to the decrease in recall.

During the training phase, we conducted a study to evaluate the influence of four parameters. Through data analysis, the objective was to discern the contribution of each module to the network’s overall functionality. Following this, we used six different hyperparameter sets to train the models multiple times. The average performance metrics from these rounds of training helped determine the best parameters for our model. Details of these hyperparameter configurations and their comparative evaluation are provided in Table 3.

**Figure 10:**
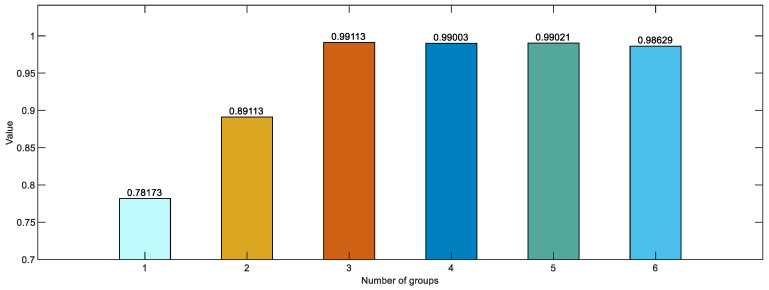
The *F*1-*score* values of experiments for each hyperparameter setting.

Following the specified hyperparameter settings, six groups underwent training and testing, with their test outcomes depicted in Figure 10.

### 5.4. Experimental Outcomes

**Figure 11:**
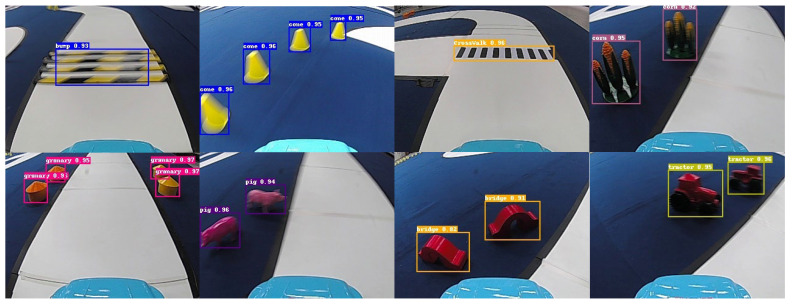
Examples of the recognition results.

In this study, we employed advanced offline data augmentation techniques as a strategic approach to enhance the model’s capability to accurately distinguish between eight distinct categories. This method involves artificially expanding the dataset with variations in the original images, thereby enabling the model to learn from a broader spectrum of instances and improve its generalization performance. The augmented data played a pivotal role in refining the model’s recognition accuracy, contributing to a more robust and adaptable object detection system. The outcomes of this experiment, which underline the effectiveness of the applied data augmentation strategies, are systematically compiled and visually depicted in Figure 11. Here, the recognition results of the tested images are carefully illustrated, offering convincing proof of the model’s improved capability in precisely recognizing and categorizing a wide range of categories.

### 5.5. Comparison Experiments

This section showcases the comparative analysis between the innovative PP-YOLOE+ model and its predecessor, PP-YOLOE, assessing metrics like accuracy, regression convergence speed, and peak frame rates. The processing speeds of each model are gauged by their frame rates.

Experiments, set in brightly lit real-world environments and depicted in Table 4, reveal that the PP-YOLOE+ model surpasses the PP-YOLOE model in maximum accuracy by 2.4%. The incorporation of depth filtering has expedited the training convergence of the PP-YOLOE+ model by 3.75-fold. Additionally, the PP-YOLOE+ model exhibits a 9.1% improvement in *AP* accuracy over the PP-YOLOE model, attaining a processing speed of 192 frames per second on a single V100 test unit, compared with the 78 FPS of the PP-YOLOE model. With mixed-precision training, the PP-YOLOE+ model achieves an inference speed that is about 105% faster than its predecessor, making it not only faster but also more accurate than competing algorithms. This improvement showcases the model’s exceptional robustness.

### 5.6. Model Evaluation

**Figure 12:**
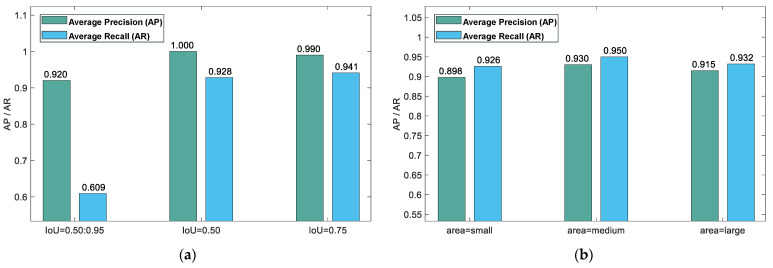
Model evaluation diagram: (**a**) Mean average precision (*mAP*) across varied IoU thresholds. (**b**) Mean average precision (*mAP*) across varied area thresholds.

Model performance is evaluated using the validation set, as depicted in Figure 12. This assessment specifically targets areas for improvement by determining the model’s predictive accuracy across different object sizes, among other insights. The evaluation criteria include the following:

(1) Calculating *mAP* across ten distinct IoU thresholds, spanning from 0.5 to 0.95 in steps of 0.05, and averaging them to obtain the *AP* measure according to the COCO dataset standard.
(2) Computing *AP* with an IoU benchmark of 0.5, corresponding to the evaluation standard of the PASCAL VOC dataset.
(3) Evaluating *mAP* with an IoU cutoff of 0.75, reflecting a more rigorous assessment due to the increased necessary overlap between the forecasted and true bounding boxes.
(4) Determining *mAP* for small (area < 32^2^), medium (32^2^ < area < 96^2^), and large objects (area > 96^2^) to evaluate model performance across object sizes.
(5) Calculating the average recall (*AR*) with a limit of 1, 10, and 100 bounding rectangles per image, which demonstrates the model’s recall capability.
(6) Calculating mean average recall (*mAR*) for small, medium, and large objects, offering insight into the model’s recall efficiency across different object scales.

This comprehensive analysis clarifies the model’s predictive performance, identifying specific areas for refinement in detecting and recognizing objects of varying sizes under distinct conditions. The methodology employed for these calculations reveals the model’s detailed ability to navigate the complexities of object detection, emphasizing the significance of precision in scenarios where object size and environmental conditions vary greatly. The analysis not only benchmarks the model against established datasets like COCO and PASCAL VOC but also extends the evaluation to incorporate the dynamic real-world applicability of the model, especially in detecting small to large objects. The insights gained from this evaluation are instrumental in guiding future enhancements of the model, ensuring that its development is aligned with the demands of practical deployment scenarios. Through meticulous assessment and targeted improvements, this model is poised to set new standards in object detection, combining high accuracy with robust performance across a spectrum of challenges.

Through the incorporation of the PP-YOLOE+ model with our dataset, the use of DIoULoss for extensive testing, and the modification of the data augmentation threshold range, we substantially improved the model’s performance upon final deployment. This strategy significantly improves the efficiency of the model, making the final test score as high as 0.99113 and the accuracy as high as 0.99786, and the detection frame rate can reach 192 FPS.

**Figure 13:**
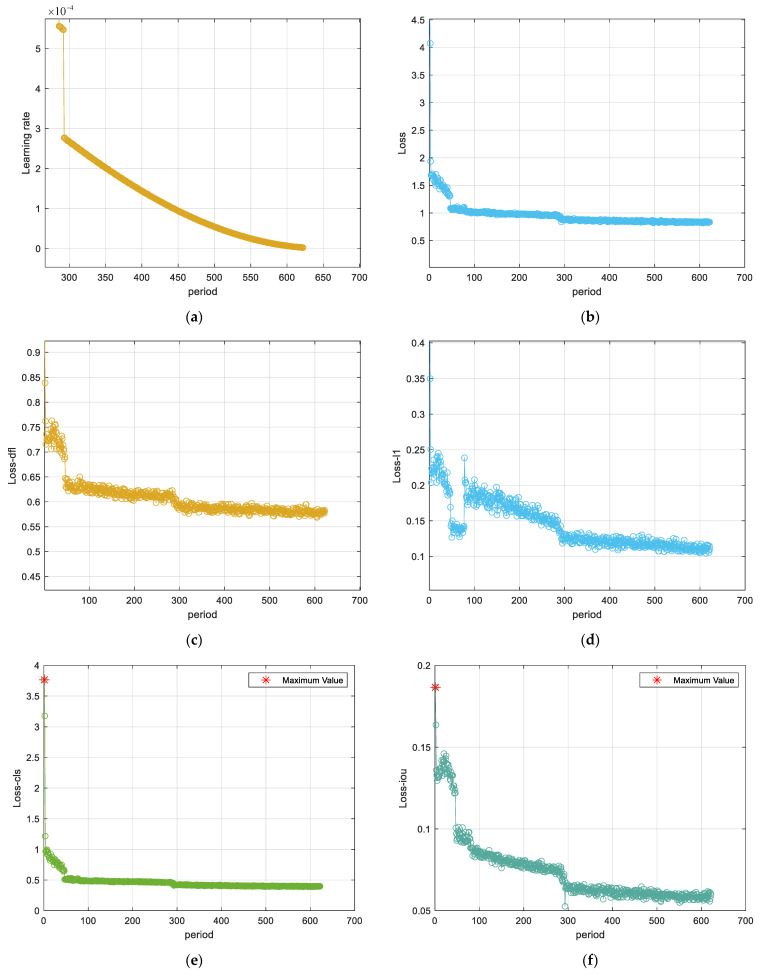

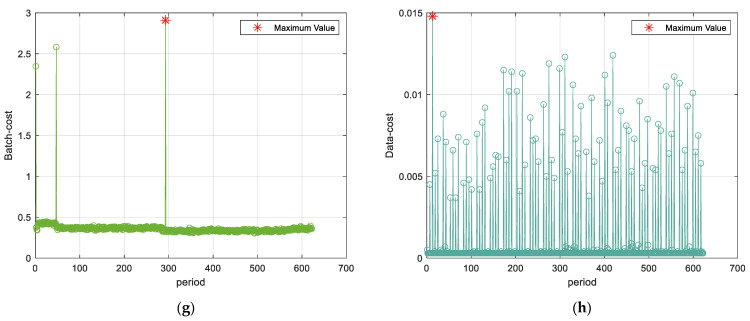
Comprehensive convergence analysis of the training for the PP-YOLOE+ model: (**a**) Convergence diagram of learning rate. (**b**) Convergence diagram of Loss. (**c**) Convergence diagram of Loss-dfl. (**d**) Convergence diagram of Loss-L1. (**e**) Convergence diagram of Loss-cls. (**f**) Convergence diagram of Loss-iou. (**g**) Convergence diagram of Batch-cost. (**h**) Convergence diagram of Data-cost.

To clarify the training performance of the PP-YOLOE+ model, Figure 13 presents the learning rate, loss, and cost metrics across the training timeline. Loss values swiftly converge to a minimal figure, with the learning rate escalating to above 0.00098 in fewer than seven epochs. Afterward, from the seventh epoch, the reduction in loss and enhancement of the learning rate slows, stabilizing as the training concludes.

### 5.7. Discussion

Based on the outcomes of our experiments, the network we developed distinguishes itself significantly from previous networks in both detection speed and accuracy, demonstrating its capability for high-precision, rapid target detection on the road. This advancement is crucial for applications requiring real-time data processing, such as autonomous driving and traffic management systems. The integration of our network into existing network layers not only allows for an expansion in the temporal dimension but also facilitates the accurate prediction and storage of sensor data. This is instrumental in enhancing the system’s ability to achieve more precise targeting and navigation, a key factor for both safety and efficiency in autonomous vehicle technologies.

Our evaluation systematically evaluates the proposed network enhancements, focusing on detection accuracy, speed, *mAP*, and FPS, which directly reflect the model’s complexity and efficiency. The findings from our study highlight the effectiveness of the individual modules and their combined integration within the network architecture. These results validate the innovative and effective approach taken in designing the network structure, highlighting the balance achieved between design efficiency and performance preservation. Through this careful balancing act, we have maintained the integrity of convolutional operations essential for deep-learning-based image analysis, without compromising on speed or accuracy.

Furthermore, this paper demonstrates the network’s ability to achieve significant lightening by leveraging image processing techniques on images captured with EdgeBoard and TC264. This approach not only enables efficient feature storage and reuse, enhancing the network’s operational efficiency but also maintains a high level of predictive accuracy. This level of precision is crucial for the network’s practical deployment in real-life situations, where the dependability of detection and prediction plays a vital role in influencing decisions and ensuring safety.

## 6. Conclusions and Future Work

In this research, we undertook an extensive optimization of the object detection component in an intelligent vehicle system, utilizing the EdgeBoard platform and the advanced PP-YOLOE+ network model.

(1) Innovation in model architecture: The PP-YOLOE+ model introduces the innovative RepResBlock, integrating residual and dense connections within both the backbone and neck of the architecture. This integration, along with the addition of the ESE modules at each stage, significantly enhances the efficiency of training deep neural networks. The enhancements lead to more sophisticated feature representation and deeper learning capabilities, which are essential for effectively processing complex visual data.

(2) Advancements in learning strategy: The implementation of the SimOTA assignment strategy alongside task-aligned learning (TAL) represents a substantial advancement in our model. By building on dynamic label assignment and task-aligned losses, TAL synchronizes classification and bounding box precision tasks. This synchronization allows for simultaneous optimization, achieving superior accuracy in both classification and localization precision.

(3) Efficiency and detection capabilities: The efficient task-aligned head (ET-head) in our model markedly enhances the system’s rapid convergence and stability, alongside improving accuracy in detecting small objects. These technical enhancements collectively forge a robust system capable of precise object detection across complex environments.

(4) Validation through rigorous testing: Our extensive experimental evaluations, conducted with our specially designed dataset, have rigorously tested the robustness and generalization performance of the PP-YOLOE+ model. The findings from these evaluations confirm that our model exceeds performance benchmarks, demonstrating exceptional effectiveness in diverse and challenging scenarios.

The evolution of intelligent vehicle systems represents an ongoing endeavor towards achieving unparalleled excellence, particularly in the realm of object detection where the escalating complexity of road environments poses formidable challenges. This landscape necessitates not only continuous algorithmic enhancements but also a forward-looking approach to research and development. In future work, we aim to delve deeper into the analysis and utilization of video data, striving for improvements in image clarity and the pursuit of highly efficient object detection mechanisms suitable for mobile implementations. The encouraging outcomes of this study underscore its potential impact on practical applications, offering robust technical foundations for the next generation of intelligent vehicles.

Our tailored mainboard and drive board, coupled with the innovative Pos-PID control method, significantly enhance the navigation precision and adaptability of autonomous vehicles, enabling them to navigate complex environments with improved accuracy and reliability. Looking ahead, our focus will be on refining our models further, particularly to excel in datasets tailored for intricate small target detection scenarios, with the goal of bolstering the system’s adaptability to complex and varied traffic environments. Moreover, we anticipate extending the methodology’s application scope to encompass a wider array of autonomous driving processes, thus making a significant contribution to the wider domain of intelligent transportation systems. This endeavor will not only push the boundaries of current technologies but also pave the way for innovative solutions in autonomous driving, enhancing safety, efficiency, and overall user experience.

We acknowledge the limitations imposed by current hardware, and our future work will be focused on investigating potential hardware enhancements and software optimizations that could support advanced versions of the PP-YOLOE+ model. Prioritizing the resolution of compatibility issues will be crucial for boosting our model’s performance. Such improvements are vital to ensure that our advancements in autonomous driving technologies keep pace with the growing demands of intelligent transportation systems, ultimately enhancing the potential of these technologies to revolutionize everyday transportation.

## Figures and Tables

**Table 1:** Overview of relevant research in intelligent vehicle object detection.

Algorithms	Advantages	Disadvantages
CNN-SSD [13]	Introduce variability convolution	Complexity of degree calculation
YOLOv4 [23]	Hollow convolution; ULSAM; soft-NMS	High computational resource
YOLO [24]	Combining R-FCN and histograms	Low parameter detection accuracy
AP-SSD [25]	Gabor feature extraction; SSD enhancement	Computational complexity
YOLOv3 [26]	Lightweight object detection framework	Poor visual effect
MAP [14]	Fading memory estimation	Low robustness complexity
CNN [15]	Multiclass object detection classifier	Low detection rate
VELIE [27]	Combining the integrated U-Net of Swin Vision Transformer and gamma transform	Gaps in detail enhancement
IDOD-YOLOv7 [28]	Combined AOD and SAIP; high accuracy	Poor practice results
Range-layer CNN [16]	High detection speed and low cost	Lack safety and reliability in autonomous driving
EYOLOv3 [29]	Kalman filter and particle filter; high efficiency	Large amount of data
SSD [30]	Structure, training method, and loss function	Suboptimal detection performance

**Table 2:** Comparison between PP-YOLOE and PP-YOLOE+.

Feature	PP-YOLOE	PP-YOLOE+
Backbone	CSPRepResStage	CSPRepResNet and new CSPPAN structure
Dynamic Label Assignment	Basic dynamic label assignment	Advanced TAL for optimized classification and localization
Detection Head	ET-head with layer attention and basic alignment modules	ET-head replaced layer attention with ESE block, and more efficient alignment modules

**Table 3:** Hyperparameter settings of different groups.

Number	Epoch	Momentum	Learning Rate	Trainreader Batch Size
1	80	0.85	0.001	8, 2, 1
2	100	0.95	0.00095	8, 4, 1
3	150	0.90	0.0009	8, 4, 2
4	180	0.95	0.001	4, 2, 1
5	250	0.90	0.0011	4, 4, 1
6	300	0.95	0.00088	4, 4, 2

**Table 4:** Results of comparison experiment on different models.

Metric	PP-YOLOE	PP-YOLOE+
mAP (%)	52.5	54.9
AP accuracy (%)	50.5	59.6
FPS	78	160
Convergence Time (h)	16	4

## Data Availability

Data are contained within the article.
